# Anti-HER2 therapy response assessment for guiding treatment (de-)escalation in early HER2-positive breast cancer using a novel deep learning radiomics model

**DOI:** 10.1007/s00330-024-10609-7

**Published:** 2024-02-08

**Authors:** Yiwei Tong, Zhaoyu Hu, Haoyu Wang, Jiahui Huang, Ying Zhan, Weimin Chai, Yinhui Deng, Ying Yuan, Kunwei Shen, Yuanyuan Wang, Xiaosong Chen, Jinhua Yu

**Affiliations:** 1 Department of General Surgery, Comprehensive Breast Health Center, Ruijin Hospital, Shanghai Jiao Tong University School of Medicine, 197 Ruijin Er Road, Shanghai, 200025 China; 2 School of Information Science and Technology, Fudan University, No. 220, Handan Road, Yangpu District, Shanghai, 200433 China; 3 Department of Radiology, Ruijin Hospital, Shanghai Jiao Tong University School of Medicine, Shanghai, 200025 China; 4 Department of Radiology, Shanghai Ninth People’s Hospital, Shanghai Jiao Tong University School of Medicine, Shanghai, 200025 China

**Keywords:** Breast cancer, Deep learning, HER2, Magnetic resonance imaging, Molecular targeted therapy

## Abstract

**Objectives:**

Anti-HER2 targeted therapy significantly reduces risk of relapse in HER2 + breast cancer. New measures are needed for a precise risk stratification to guide (de-)escalation of anti-HER2 strategy.

**Methods:**

A total of 726 HER2 + cases who received no/single/dual anti-HER2 targeted therapies were split into three respective cohorts. A deep learning model (DeepTEPP) based on preoperative breast magnetic resonance (MR) was developed. Patients were scored and categorized into low-, moderate-, and high-risk groups. Recurrence-free survival (RFS) was compared in patients with different risk groups according to the anti-HER2 treatment they received, to validate the value of DeepTEPP in predicting treatment efficacy and guiding anti-HER2 strategy.

**Results:**

DeepTEPP was capable of risk stratification and guiding anti-HER2 treatment strategy: DeepTEPP-Low patients (60.5%) did not derive significant RFS benefit from trastuzumab (*p* = 0.144), proposing an anti-HER2 de-escalation. DeepTEPP-Moderate patients (19.8%) significantly benefited from trastuzumab (*p* = 0.048), but did not obtain additional improvements from pertuzumab (*p* = 0.125). DeepTEPP-High patients (19.7%) significantly benefited from dual HER2 blockade (*p* = 0.045), suggesting an anti-HER2 escalation.

**Conclusions:**

DeepTEPP represents a pioneering MR-based deep learning model that enables the non-invasive prediction of adjuvant anti-HER2 effectiveness, thereby providing valuable guidance for anti-HER2 (de-)escalation strategies. DeepTEPP provides an important reference for choosing the appropriate individualized treatment in HER2 + breast cancer patients, warranting prospective validation.

**Clinical relevance statement:**

We built an MR-based deep learning model DeepTEPP, which enables the non-invasive prediction of adjuvant anti-HER2 effectiveness, thus guiding anti-HER2 (de-)escalation strategies in early HER2-positive breast cancer patients.

**Key Points:**

• *DeepTEPP is able to predict anti-HER2 effectiveness and to guide treatment (de-)escalation.*

• *DeepTEPP demonstrated an impressive prognostic efficacy for recurrence-free survival and overall survival.*

• *To our knowledge, this is one of the very few, also the largest study to test the efficacy of a deep learning model extracted from breast MR images on HER2-positive breast cancer survival and anti-HER2 therapy effectiveness prediction.*

**Supplementary Information:**

The online version contains supplementary material available at 10.1007/s00330-024-10609-7.

## Introduction

Breast cancer is the most common malignancy in women worldwide [1]. The incidence of breast cancer in China is increasing rapidly, which has severely endangered women’s life and health [2, 3]. The emergence of anti-human epidermal growth factor receptor 2 (HER2) agents has significantly improved survival of HER2-positive breast cancer patients, which accounts for 15–20% of breast cancer population [4–8]. Optimizing anti-HER2 strategy to maximize drug efficacy and minimize treatment-related toxicities has become a significant challenge for physicians. There is currently an unmet need for better risk stratification in these HER2-positive breast cancer patients, to identify high risk population for anti-HER2 escalation, and to spare low risk patients from unnecessary treatment.

In current clinical practice, HER2 testing was accomplished on tumor samples according to international guidelines [9]. Immunohistochemistry (IHC) and fluorescence in situ hybridization (FISH) are the two primary techniques for the determination of HER2 status [9]. Nevertheless, due to relatively limited tissue sampling, tumor spatial and temporal heterogeneity may render these tissues less representative for the entire tumor [10]. As a supplement, radiographic imaging, such as magnetic resonance (MR) imaging, offers morphological and functional information with an overall sensitivity of 98–100% and a specificity of 88% for breast malignancy diagnosis [11]. With kinetic or dynamic enhancement assessment, MR showed superior capacity in local cancer staging, high-risk patient screening, and disease extent identification [12–14].

In recent decades, radiomics has become an important direction in medical image analysis. It treats medical images as minable data to build models for clinical diagnosis, treatment plan selection, and prognosis prediction [15, 16]. Due to the superior performance of deep learning, it has become an indispensable technique in radiomics modeling [17]. As a class of machine learning algorithms, deep learning constructs networks capable of learning from unstructured data. Unlike conventional machine learning methods, for example linear regression, Naïve Bayes classifier, or support vector machines (SVM), deep learning algorithms recruit multiple layers to extract high-throughput features [18, 19]. Previous deep learning studies demonstrated high sensitivity for breast cancer diagnosis. Abdel-Zaher et al developed a deep belief network unsupervised path followed by back propagation supervised path, which showed an accuracy of 99.68% for breast cancer detection [20]. A mammogram-based, semi-supervised learning with convolution neural network showed a sensitivity of 81% and a specificity of 72% [21]. In addition to breast cancer diagnosis, deep learning has also been applied in disease staging, as well as response evaluation. A clinical parameter-combined, ultrasound-based deep learning radiomics model reported an area under the receiver operating characteristic curve (AUC) of 0.902 in identifying metastatic axillary lymph node [22]. Another cohort using Inception V3 deep learning model achieved an AUC of 0.89 in the prediction of nodal metastasis from clinical node-negative patients [23]. The deep learning algorithm from Qu et al attained an AUC of 0.968 for pathological response prediction to neoadjuvant chemotherapy in breast cancer by a comprehensive analysis of pre- and post-treatment images [24]. Notably, breast MR consists of multiple layers of images with varying signal intensities and tissue contrast, which brings more challenges for the implementation of deep learning algorithms [25–27]. So far, very few studies were powered to show a prognostic value of deep learning model in predicting clinical outcomes of breast cancer patients. Furthermore, there is currently no radiomics model capable of providing anti-HER2 treatment strategy guidance.

To that end, here we developed a novel deep learning algorithm based on preoperative breast MR imaging, DeepTEPP (Deep-learning-based Treatment Effectiveness and Prognosis Predictor), to predict anti-HER2 treatment response and more importantly, to guide the escalation or de-escalation of anti-HER2 therapy.

## Materials and methods

### Study population and MR acquisition

**Fig. 1:**
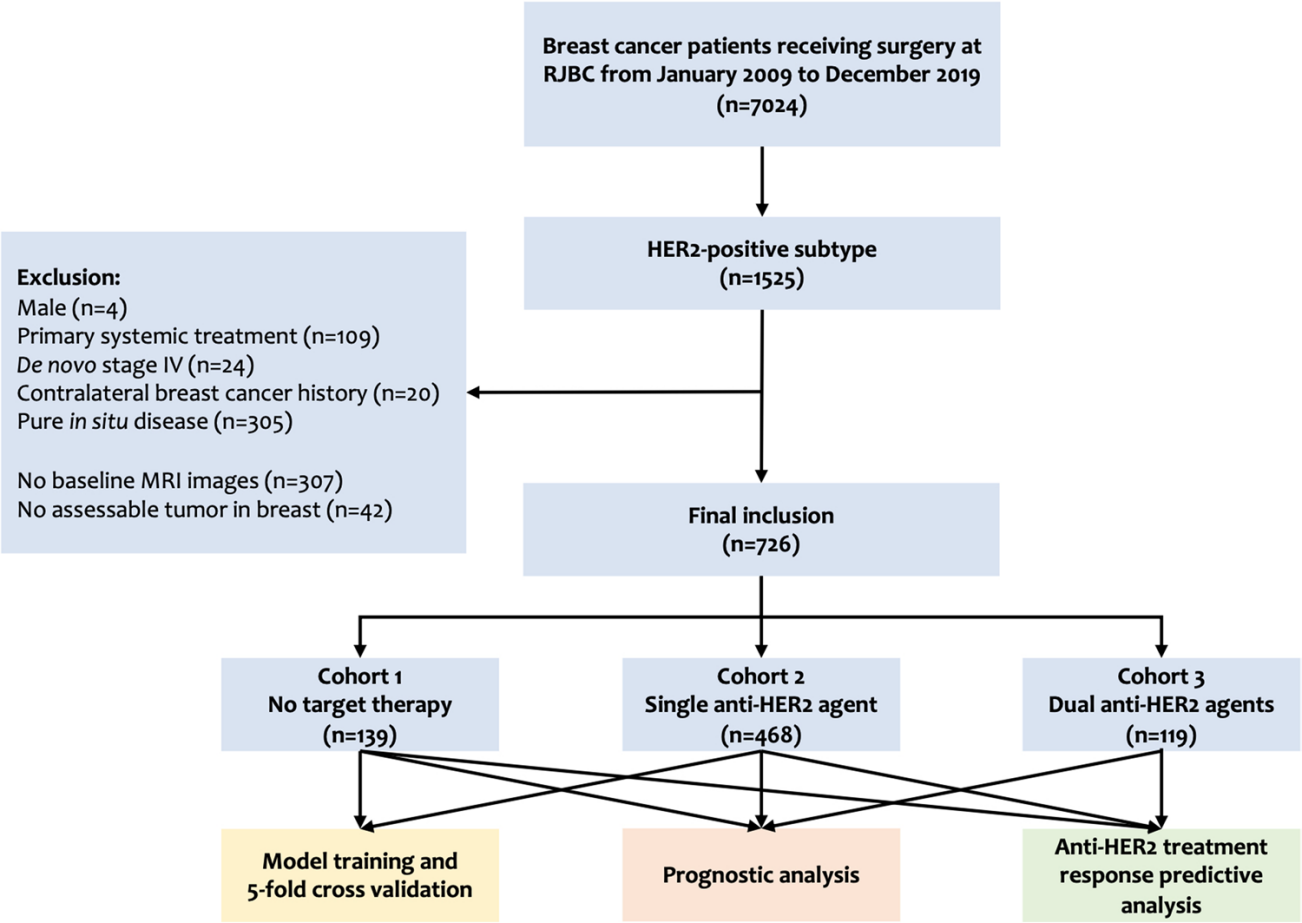
Study flowchart. Abbreviations: HER2, human epidermal growth factor receptor-2; MRI, magnetic resonance imaging

Consecutive breast cancer patients surgically treated in the Comprehensive Breast Health Center, Ruijin Hospital, Shanghai Jiao Tong University School of Medicine, between January 2009 and December 2017 were retrospectively included (Fig. 1). The eligibility criteria were as follows: (1) female gender; (2) complete sequences of breast-specific MR images prior to any invasive procedure; (3) histologically proven invasive breast cancer; (4) HER2-positive disease, defined as IHC 3 + or FISH-positive according to the 2018 ASCO/CAP guidelines [9]; (5) complete clinical-pathological (CP) features; (6) complete follow-up. Those who received biopsy or surgery before MR, who received primary systemic treatment, with de novo stage IV diseases, or with no assessable tumor in the breast were excluded.

Overall, a total number of 726 out of 7024 breast cancer patients were included and split into three respective cohorts according to the adjuvant anti-HER2 treatment they received: cohort 1, no target therapy (*N* = 139); cohort 2, single agent target therapy with trastuzumab (*N* = 468); cohort 3, dual blockade with trastuzumab and pertuzumab (*N* = 119; Fig. 1). Detailed clinical pathological, treatment, and follow-up information were retrieved from Shanghai Jiao Tong University Breast Cancer Database (SJTU-BCDB), as presented in Table 1. After a median follow-up of 64.2 (range 14.0–124.0) months, 60 (8.26%) disease relapses were reported in the whole cohort, including 15 loco-regional recurrences, 13 contralateral breast cancer, and 32 distant metastasis. Thirteen patients deceased from breast cancer (Table S1).

**Table 1:** Clinical-pathological features of patients

Variables	Total *N* = 726	No target therapy *N* = 139	T *N* = 468	T + P *N* = 119
Age, year				
$<$ 50	235	43	160	32
50–65	414	70	269	75
$>$ 65	77	26	39	12
Menstruation status				
Pre-/peri-	262	47	179	36
Post-	464	92	289	83
Breast surgery				
BCS	142	26	102	14
Mastectomy	584	113	366	105
Axillary surgery				
SLNB	291	53	219	19
ALND	435	86	249	100
Tumor size, cm				
$\le$ 2	338	64	230	44
$>$ 2	388	75	238	75
Node status				
Negative	365	85	270	10
Positive	361	54	198	109
Stage				
I	205	47	158	0
IIA	245	48	158	39
IIB	126	25	66	35
IIIA	90	9	52	29
IIIC	60	10	34	16
Nuclear grade				
I–II	242	51	158	33
III	484	88	310	86
ER status				
Negative	391	65	264	62
Positive	335	74	204	57
PR status				
Negative	511	91	340	80
Positive	215	48	128	39
Ki-67, %				
$<$ 14	63	17	37	9
$\ge$ 14	663	122	431	110
Molecular subtype				
HR-positive	335	74	204	57
HR-negative	391	65	264	62

*T* trastuzumab, *P* pertuzumab, *BCS* breast conserving surgery, *SLNB* sentinel lymph node biopsy, *ALND* axillary lymph node dissection, *ER* estrogen receptor, *PR* progesterone receptor, *HR* hormone receptor

MR, including three-dimensional T1-weighted and T2-weighted, magnetic resonance spectroscopy, and diffusion-weighted imaging, was performed with breast-specific MR imager (MAGNETOM Aera; Siemens Healthcare or Aurora; Aurora Healthcare) with a four-channel bilateral breast coil in the axial orientation prior to any invasive procedure in the Department of Radiology of Ruijin Hospital by experienced radiologists. Dynamic contrast-enhanced (DCE) images were obtained as five post-injection scans with intervals of 30 s following the intravenous injection of a gadolinium-based agent using the following scan parameters: TR/TE 4.5/1.6 ms, field of view 340 × 340 mm^2^, matrix 384 × 385, flip angle 10°, slice thickness 1.0 mm, number of slices 104, and total duration of T1-weighted imaging 450 s. The third phase, where the most apparent enhancement was observed, was selected for automatic tumor detection and regions of interest (ROIs) extraction.

This study was approved by the independent Ethical Committees of Ruijin Hospital, Shanghai Jiao Tong University School of Medicine (approval code: 2020–309; date of approval: 17 September 2020). All human-related procedures were in conformity with the 1964 Helsinki declaration and its later amendments, with the ethical standards of the national research committee. At the time of clinical examinations, patients provided written informed consent for use of anonymized data in any future retrospective research.

### Network architecture

**Fig. 2:**
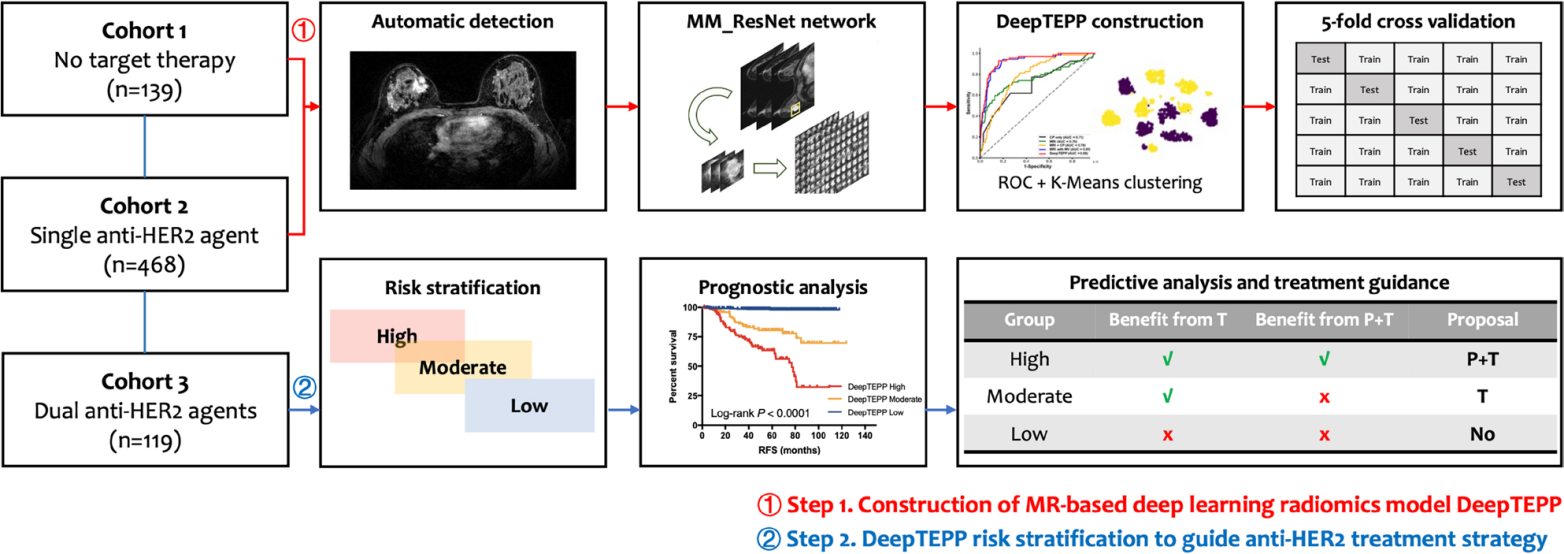
Schematic outline for DeepTEPP model construction and validation. DeepTEPP was composed of two steps: step 1 is to build a deep learning scoring system through automatic tumor detection, multi-view data augmentation, jigsaw shuffle, network training, clinicopathological factors fusion, and unsupervised K-Means clustering. The predictive value of the model was evaluated using the AUC of the ROC curve over fivefold cross-validation. Step 2 is the risk stratification based on DeepTEPP scoring in cohorts 1, 2, and 3, where the extent to which the patients in each risk group benefit from anti-HER2 treatment were analyze and treatment strategy was proposed. Abbreviations: HER2, human epidermal growth factor receptor-2; ROC, receiver operating characteristic; RFS, recurrence-free survival; T, trastuzumab; P, pertuzumab; AUC, area under curve

The construction and validation of DeepTEPP was based on data from cohorts 1 and 2 (Fig. 2, step 1). Patient recurrence status is set as the learning ground truth. Recurrence events include invasive ipsilateral and local/regional recurrence, distant metastasis in any site, and death from breast cancer. Key techniques for DeepTEPP are automatic tumor detection (Figure S1), multi-view (MV) data augmentation [28–31] (Figure S2A), jigsaw shuffle [32, 33] (Figure S2B-C), MM_ResNet (multi-scale and multi-stage improved ResNet) network training [34] (Figure S2D), and CP fusion. Breast tumor detection is the premise of the system for further tumor feature extraction. After detection, we designed a novel multi-view data augmentation to balance the proportion of positive and negative samples. The jigsaw shuffle strategy used slice splicing and shuffle filtering to ensure that the network could extract inter-layer contextual information. Building on the previous steps, we introduce a network MM_ResNet that extracts multi-layered as well as multi-scale features from MR images. Finally, CP information is introduced to fuse into the MM_ResNet, and the probability value of the last full connection layer after the softmax activation function is taken as the risk score. The softmax function is to produce a probability distribution over labels such that most of the mass is situated at the maximum entry of the output vector. If the classifier is very confident about the output, then the corresponding risk score should be close to 1. Patients scoring below the cutoff determined by the highest Youden Index cutoff point are defined as low risk. Patients scoring above the cutoff are further classified into moderate- and high-risk groups using unsupervised K-Means clustering. The predictive value of the model was evaluated using the AUC of the receiver operating characteristic (ROC) curve over fivefold cross-validation on cohorts 1 and 2. Detailed steps of network architecture are presented in Supplementary File S1.

### Experimental design

To validate the effectiveness of our proposed method, we designed three experiments as follows. First, effectiveness comparison: We conducted a comprehensive investigation involving the exploration of different network backbones, clinical indicators, and relevant ablation experiments where we compared the effectiveness of different configurations, including the baseline using MR alone (MM_ResNet), CP alone, MR + CP, MR + MV, and our proposed method DeepTEPP (MR + CP + MV) using ROC curves (Fig. 2, step 1). Second, verification of the treatment guiding value of DeepTEPP by comparing clinical outcomes of patients receiving different treatments in each risk group. Thirdly, validation of the prognostic value of DeepTEPP by using Kaplan–Meier curve with log-rank tests (Fig. 2, step 2).

### Statistical analysis

In step 1, ROC curve is applied to compare the predictive performance of different networks. Sensitivity is defined as true positive rate, where a known positive condition is predicted positive, while specificity is defined as true negative rate, where a known negative condition is predicted negative. The cutoff value with the highest Youden Index or, equivalently, the highest sensitivity plus specificity was adopted for the identification of low-risk individuals in step 1 [35].

In step 2, propensity score matching (PSM) was applied to match patients treated with trastuzumab + pertuzumab versus trastuzumab according to tumor stage and follow-up time. Clinical outcomes between different risk categories, including recurrence-free survival (RFS) and overall survival (OS), were compared using Kaplan–Meier curve with log-rank tests. RFS was calculated from surgery to the first proven recurrent event including invasive ipsilateral and local/regional recurrence, distant metastasis in any site, and death of any cause. OS was calculated from surgery till the date of death from any cause. Subgroup interaction analysis on the prognostic value of the selected model was conducted by using the stratified Mantel–Haenszel test to estimate the hazard ratio (HR) with a 95% confidence interval (CI). All statistical analyses were performed using R packages version 3.4.2 (https://cran.r-project.org/), and Python version 3.6. GraphPad Prism version 7.0 was applied in image production. Two-sided *p* < 0.05 was considered statistically significant.

## Results

### Comparative analysis of different backbones and ablation experiments

In order to find the most suitable base network for recurrence status prediction tasks, the performances of VGG16 [36], Desnet169 [37], and our proposed network (Shake [38], SE_ResNeXt [39], and MM_ResNet) were compared (Table S2). The MM_ResNet achieved the highest AUC of 0.76 in the fivefold cross-validation, and an AUC of 0.65 in the independent testing set, significantly higher than that of other methods including VGG16 (AUC, 0.51), Desnet169 (AUC, 0.62), Shake (AUC, 0.61), and SE_ResNeXt (AUC, 0.58), and was thus chosen as network backbone. It is noteworthy that the specificity of our network (96.65%) was also universally better than other methods. Univariate analysis demonstrated that tumor size stage, lymph node status, estrogen receptor status, progesterone receptor status, and molecular subtype were associated with RFS (all *p* < 0.05; Table S3), and were included as CP features to construct the DeepTEPP scoring network.

The DeepTEPP model achieved the highest AUC of 0.88 in predicting recurrence, greatly outperforming MR alone, CP alone, and MR + CP, whose AUC was 0.76, 0.71, and 0.78 respectively (all *p* < 0.001; Table 2; Figure S3A). Moreover, it also had a numerical improvement on the basis of MR + MV (AUC 0.85, *p* = 0.297). In the independent testing set, the AUC of DeepTEPP reached 0.87, which was significantly higher than the AUC of CP only, MR only, and MR + CP (0.73, *p* < 0.001; 0.65, *p* < 0.001; and 0.74, *p* = 0.022; Table 2), similar to MR + MV (AUC 0.86, *p* = 0.286). When stratified by trastuzumab treatment, the AUC of the trastuzumab-treated cohort was slightly higher than that of the non-trastuzumab cohort, both cohorts achieving satisfactory classification efficiency (0.94 *vs* 0.93; Figure S3B), suggesting the robustness of DeepTEPP model.

**Table 2:** Performance summary of different models in predicting recurrence-free survival for HER2-positive patients

Models	Dataset	AUC	ACC (%)	SPEC (%)	SENS (%)
CP only	CV	0.71	87.49	97.21	11.59
	T	0.73	85.25	93.52	21.43
MRI	CV	0.76	89.79	96.65	36.23
	T	0.65	74.77	87.04	42.86
MRI + CP	CV	0.78	80.23	84.20	49.28
	T	0.74	82.79	88.89	35.71
MRI with MV	CV	0.85	84.35	85.51	84.20
	T	0.86	85.45	75.00	96.30
DeepTEPP	CV	0.88	85.01	91.30	84.20
(MRI with MV + CP)	T	0.87	90.00	80.95	93.52

*HER2* human epidermal growth factor receptor-2, *AUC* area under curve, *ACC* accuracy, *SPEC* specificity, *SENS* sensitivity, *MRI* magnetic resonance images, *CV* cross-validation, *T* independent testing set, *CP* clinical-pathological, *MV* multi-view augmentation

Through steps 1 and 2, we stratified 439 (60.5%), 144 (19.8%), and 143 (19.7%) patients into DeepTEPP-Low, Moderate, and High groups, respectively (Fig. 2).

### DeepTEPP and anti-HER2 treatment triage

**Fig. 3:**
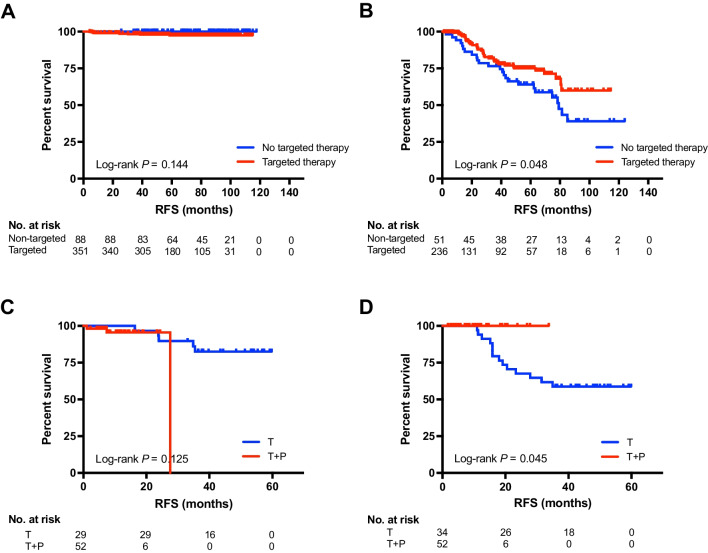
Predictive value and target therapy algorism according to DeepTEPP. **A** DeepTEPP-Low patients do not derive significant RFS benefit, while (**B**) DeepTEPP-Moderate and -High patients significantly benefit from anti-HER2 targeted therapy. **C** DeepTEPP-Moderate achieve no additional RFS benefit from pertuzumab on the basis of trastuzumab, while (**D**) DeepTEPP-High patients derived improved RFS with dual HER2 blockade compared to single agent trastuzumab. Abbreviations: RFS, recurrence-free survival; T, trastuzumab; P, pertuzumab

Afterwards, we tested the predictive value of DeepTEPP for adjuvant anti-HER2 targeted treatment. Eighty-eight DeepTEPP-Low patients did not receive anti-HER2 treatment, and the others completed 1 year trastuzumab. DeepTEPP-Low patients did not derive significant benefit from trastuzumab therapy (5-year RFS 97.4% *vs* 98.9%, *p* = 0.144; Fig. 3A; 5-year OS 98.6% *vs* 98.9%, *p* = 0.931; Figure S4A). On the other hand, 287 (39.5%) patients were categorized into DeepTEPP-Moderate or High, who significantly benefited from anti-HER2 treatment (5-year RFS 64.0% *vs* 75.0%, *p* = 0.048; Fig. 3B; 5-year OS 72.9% *vs* 91.5%, *p* = 0.001; Figure S4B). For patients with DeepTEPP-Moderate and High risk, RFS and OS were then compared between patients receiving single-agent trastuzumab and those receiving dual blockade trastuzumab + pertuzumab in each risk group after PSM by tumor stage and follow-up time. DeepTEPP-Moderate patients did not obtain additional improvements from the addition of pertuzumab to trastuzumab (2y-RFS 89.7% *vs* 95.4%, *p* = 0.125; Fig. 3C; 2y-OS 96.6% *vs* 100.0%, *p* = 0.746; Figure S4C). Meanwhile, DeepTEPP-High patients receiving dual HER2 blockade had substantially improved RFS compared to those treated with trastuzumab alone (2y-RFS 67.7% *vs* 100.0%, *p* = 0.045; Fig. 3D), indicating DeepTEPP was capable of risk stratification and could guide adjuvant anti-HER2 target treatment strategy in early HER2-positive breast cancer patients (Fig. 2).

### Prognostic value of DeepTEPP

**Fig. 4:**
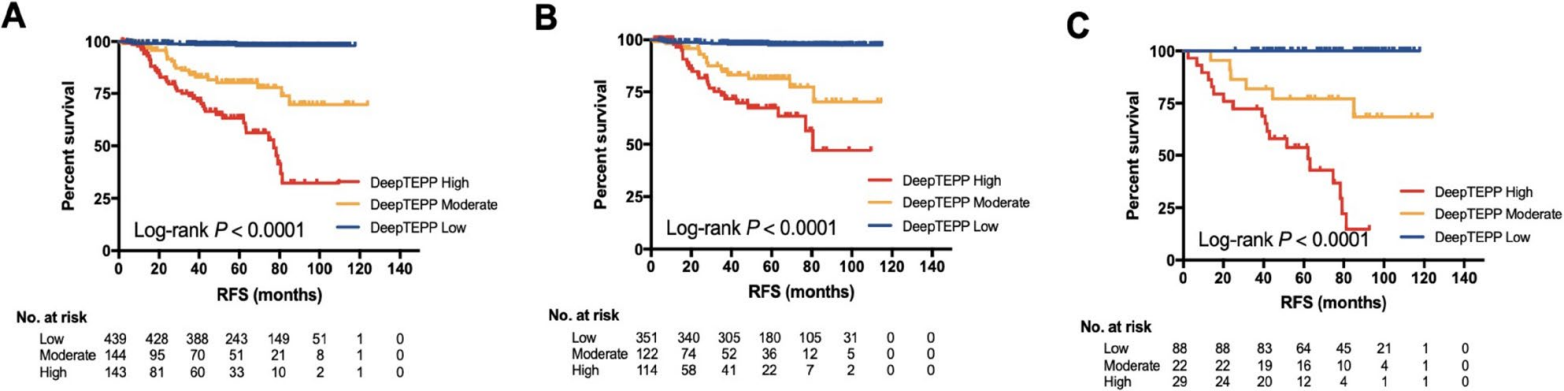
Prognostic value of DeepTEPP for recurrence-free survival. Recurrence-free survival according to DeepTEPP in the whole cohort (**A**), in patients treated with anti-HER2 targeted therapy (**B**), and in those who did not receive anti-HER2 treatment (**C**)

DeepTEPP was further tested for its prognosis predictive value. DeepTEPP-Low patients had significantly better RFS (5-year RFS 98.0%, 80.3%, 63.3%, respectively, *p* < 0.0001; Fig. 4A) and OS (5-year OS 98.7%, 90.8%, 80.5%, *p* < 0.0001; Figure S5A), compared to DeepTEPP-Moderate and High patients. Multivariate analysis demonstrated that DeepTEPP category was the strongest independent prognostic factor after adjusting for tumor stage and adjuvant anti-HER2 treatment for either RFS (Moderate *vs* Low: hazard ratio [HR] 12.22, 95% CI 5.37–27.79, *p* < 0.0001; High *vs* Low: HR 28.04, 95% CI 12.87–61.07, *p* < 0.0001) or OS (Moderate *vs* Low: HR 8.29, 95% CI 2.72–25.24, *p* < 0.0001; High *vs* Low: HR 11.17, 95% CI 3.82–32.67, *p* < 0.0001).

The prognostic value of DeepTEPP was then evaluated according to adjuvant anti-HER2 target treatment usage. For those receiving targeted therapy, both risk of recurrence and death increased with greater DeepTEPP risk (5-year RFS 97.4%, 81.4%, 67.5%, *p* < 0.0001; Fig. 4B; 5-year OS 98.6%, 94.0%, 88.1%, *p* = 0.0003; Figure S5B). In the meantime, such difference remained statistically significant for both RFS and OS in those who did not receive anti-HER2 treatment (5-year RFS 98.9%, 77.0%, 53.8%, *p* < 0.0001, Fig. 4C; 5-year OS 98.9%, 81.3%, 65.3%, *p* < 0.0001, Figure S5C).

Further subgroup analysis revealed that the prognostic value of the DeepTEPP model was consistent throughout each subgroup, with a higher score predicting worse RFS (Figure S6A) and OS (Figure S6B) in all subgroups.

## Discussion

In the current study, we built a preoperative breast MR-based deep learning network with automatic tumor detection and MV data augmentation using a cohort of 726 consecutive HER2-positive breast cancer patients receiving different kinds of anti-HER2 treatments. The so-called DeepTEPP (Deep-learning-based Treatment Effectiveness and Prognosis Predictor) network could not only predict disease outcomes, but also predict adjuvant anti-HER2 treatment effectiveness, thus to guide anti-HER2 targeted treatment strategy in early HER2-positive breast cancer patients. Our proposed DeepTEPP model demonstrated an impressive predictive efficacy for recurrence (validation cohort: AUC 0.88; testing cohort: AUC 0.87). Furthermore, DeepTEPP is to our knowledge the first known deep learning model to guide anti-HER2 (de-)escalation, providing an important reference for choosing the appropriate individualized treatment for HER2-positive breast cancer patients in a non-invasive way.

Over the past decades, various deep learning models have been proposed to extract high-dimensional data from digital medical images to help answer clinical questions. In the field of breast cancer, MR-based deep learning has been applied to predict pathologic response following neoadjuvant treatment (AUC 0.47–0.99 [24, 40, 41]), to predict axillary lymph node metastasis (AUC 0.81–0.86 [42–44]), and to identify tumor characteristics (AUC 0.80–0.85 [45, 46]). The fact is, however, that no widely spread clinical application or widely acknowledged model exists in the real-world setting. On the basis of previous evidence, our network has filled several vacancies in the field. For instance, most previous studies have tested the overall breast cancer subtypes together, while we focused on the specific HER2-positive subtype. Furthermore, very few studies were powered to construct a deep learning model, capable of prognosis prediction for cancer patients, while in the current study our DeepTEPP network was not only prognostic for RFS but also for OS. In terms of the deep neural network, most of the existing networks use classical neural networks like VGG16, ResNet [34, 36] to complete feature extraction and classification. However, these networks ignore the multi-scale, fine-grained, and multi-slice information of the inputted MR images. We solved these problems by adding a few parameters on top of ResNet. Specifically, the features of multiple network feature layers were extracted to represent the multi-scale information of the images, and the jigsaw shuffle strategy was used to extract the fine-grained and *z*-axis information. Last but not least, the operation time required for the calculation of our deep learning model was minimal, with network parameter amount costing only 45 Mb, which can be easily applied on mobile or hand-held devices, leading to possible clinical implementation.

Conventional big data-driven deep learning relies on large amounts of medical images to build intelligent diagnostic models [47]. Due to the difficulty of collecting medical data and a relatively low incidence of survival events, there are often problems such as small sample sizes [48] or unbalanced positive/negative samples [49]. Data augmentation methods are often applied to solve these problems, including flip, rotate, crop, etc. [26]. These methods are only simple mapping of the original image, so the difference between enhanced data and original data is still small which limits the diversity of data. In addition, only the axial, coronal, and sagittal images of MR were generally used in model construction, but the 3D character of the tumor was ignored. In the current study, we propose the MV data augmentation, a novel 3D data augmentation technique, to improve the accuracy of classification. In detail, our proposed method can not only solve the positive/negative sample imbalance by increasing sample size for the category with lower frequency, but also generate new data in case of a small sample size to prevent network overfitting.

Trastuzumab-based anti-HER2 treatment brings significant survival benefit compared to chemotherapy alone in HER2-positive early breast cancer patients [4–8]. Meanwhile, treatment-related adverse effects and financial toxicity also cause severe burden to both patients and health care systems. For instance, cost-effectiveness analyses of trastuzumab monotherapy showed an incremental cost-effectiveness ratio ranging from 3 to 170 thousand USD per quality-adjusted life year gained in early breast cancer patients [50]. Moreover, pertuzumab, another anti-HER2 monoclonal antibody, could further improve disease outcomes for high-risk HER2 + patients on the basis of trastuzumab and chemotherapy. It is noteworthy that the current study is one of the very few, also the largest study to evaluate not only the prognostic value, but also the predictive efficacy of deep learning model in a consecutive cohort of HER2-positive breast cancer patients. Thus, our model provides guidance to fullfill the unmet need to better stratify high-risk population for treatment escalation or de-escalation, and to spare low-risk patients from unnecessary treatment, which highlights the clinical relevance of the current study. To note, the 5-year RFS in the DeepTEPP-Low, -Moderate, and -High groups was 97.4%, 81.4%, and 67.5% in those receiving anti-HER2 treatment, respectively, demonstrating a comparable prognostic and predictive value to the multigene assay HER2DX model, which reported a 5-year DFS of 93.5%, 86.7%, and 81.1% in the HER2DX low-risk, medium-risk, and high-risk groups [51]. We believe that the combination of multigene assay and radiomic model might help better tailor systemic therapy and select patients to omit unnecessary treatment.

Nevertheless, there existed several limitations. Given the nature of the single-center design, external validation was lacking, and the robustness of our model has not been tested according to different MR machines. Prospective multi-center validation will be carried out in future studies to promote our model for clinical application. Secondly, since deep neural network training was driven and promoted by a large scale of data, the current sample size was still limited and might prevent the network from better performance. Although we partially overcome this problem by MV data enhancement and extracting multiple imaging samples from the same patient, larger patient populations may further improve algorithm performance. More interestingly, we found that CP did not significantly add power to predict survival. We fused MR and CP information by concatenating CP with feature layers in front of classifiers [20]. One possible explanation is that some of the CP features have already been extracted from the imaging data, such as tumor size and lymph node status. Therefore, the role of CP features is more about supplementing clinical information that cannot be extracted from the images alone. Moreover, here DeepTEPP was first built and trained in cohorts 1 and 2 in step 1, and cohort 3 was not used for training purposes but served as an independent testing set to predict the scores. Since pertuzumab was available for routine clinical use in our center only after 2019, the follow-up time for cohort 3 is significantly shorter compared to cohorts 1 and 2, which may introduce inaccuracies in the ground truth labels. As a result, we tested cohort 3 separately to avoid the potential bias that the inclusion of cohort 3 would bring to the deep learning model. Despite our efforts by adjusting with PSM, longer follow-up and larger cohort were necessary to better establish survival differences. Still, future efforts are warranted, with the help of multi-omics, to better understand the underlying molecular biological mechanism behind imaging phenotypes.

## Conclusions

In conclusion, we developed a novel MR-based deep learning algorithm named DeepTEPP, which can accurately predict disease outcomes as well as anti-HER2 targeted treatment benefit, thus to guide further individualized de-escalation and escalation treatment in early HER2-positive breast cancer patients.

### Supplementary Information

Below is the link to the electronic supplementary material.

Supplementary file1 (PDF 1024 KB)

## Abbreviations

AUC: Area under curve
CI: Confidence interval
DCE: Dynamic contrast-enhanced
FISH: Fluorescence in situ hybridization
HER2: Human epidermal growth factor receptor 2
HR: Hazard ratio
IHC: Immunohistochemistry
MRI: Magnetic resonance imaging
MV: Multi-view
OS: Overall survival
PSM: Propensity score matching
RFS: Recurrence-free survival
ROC: Receiver operating characteristic
ROI: Region of interest
SVM: Support vector machines

## Declarations

### Guarantor

The scientific guarantor of this publication is Jinhua Yu.

### Statistics and biometry

No complex statistical methods were necessary for this paper.

### Study subjects or cohorts overlap

No study subjects or cohort overlap has been reported.

### Methodology

• retrospective

• diagnostic or prognostic study

• performed at one institution
