# Supplementary material for: Anti-HER2 therapy response assessment for guiding treatment (de-)escalation in early HER2-positive breast cancer using a novel deep learning radiomics model
Source: Eur Radiol. 2024 Feb 8;34(8):5477–86. doi: 10.1007/s00330-024-10609-7 (PMC11255056; doi:10.1007/s00330-024-10609-7)
Supplement: Supplementary file 1 — Supplementary file1 (PDF 1024 KB) [file 330_2024_10609_MOESM1_ESM.pdf]

Figure S1. Tumor automatic detection flowchart.

Figure S2. Multi-view data augmentation, jigsaws shuffle strategies and construction of RFS classification network.

Figure S3. Predictive performance of DeepTEPP.

Figure S4. Overall survival by target therapy in different DeepTEPP risk groups.

Figure S5. Prognostic value of DeepTEPP for overall survival.

Figure S6. Subgroup analysis for the performance of DeepTEPP.

Table S1. Disease outcomes.

Table S2. Performance comparison of different networks.

Table S3. Univariate survival analysis of clinical-pathological variables.

Supplementary File S1

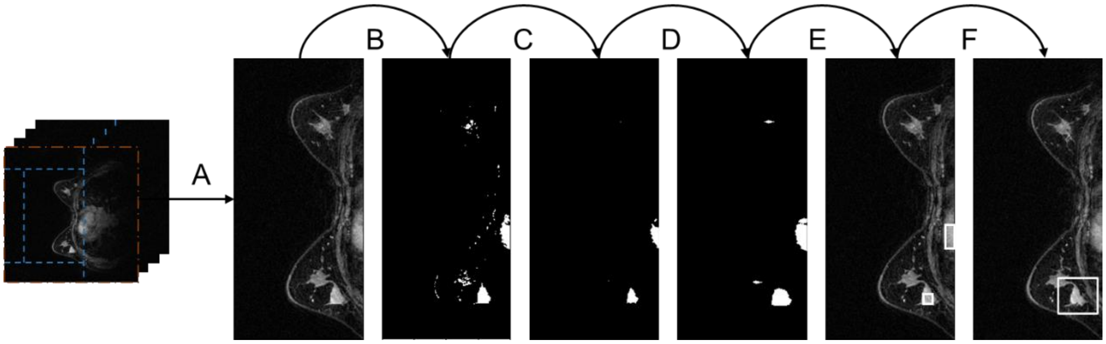

Figure S1. Tumor automatic detection flowchart.

Flowchart of automatic detection of breast tumor on magnetic resonance imaging. (A) breast region cropping; (b) binarization; (c) erosion; (d) dilation; (e) central location selection; (f) screening of tumor location.

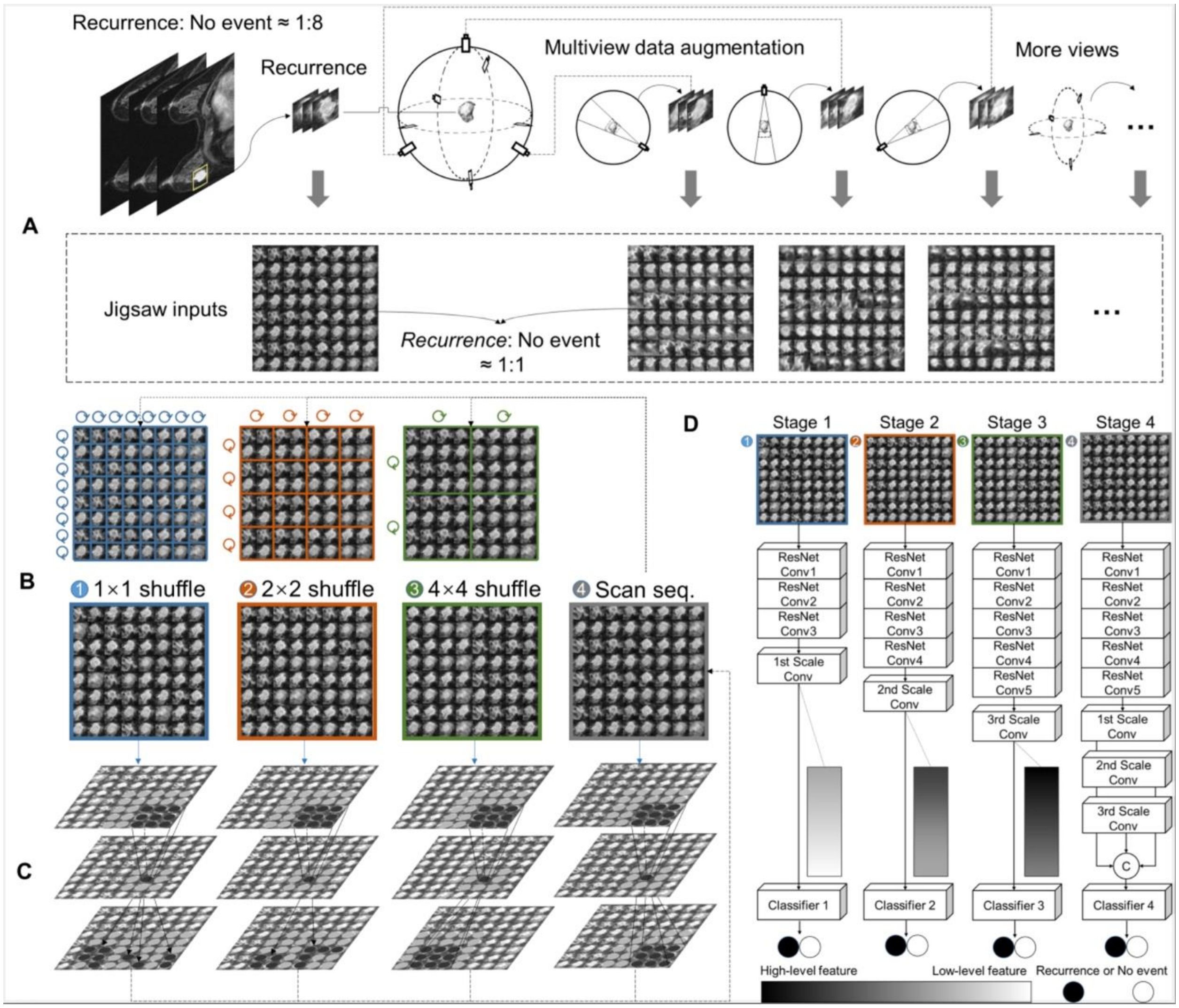

Figure S2. Multi-view data augmentation, jigsaws shuffle strategies and construction of RFS classification network.

Depiction of the deep learning based workflow after multi-view data augmentation and shuffling strategies. (A) multi-view data augmentation method; (B) strategies of shuffling jigsaws; (C) different receptive fields under different shuffle strategies; (D) the multi-scale multi-stage ResNet for RFS classification.

Abbreviations: RFS, recurrence-free survival. Scan seq., scan sequence.

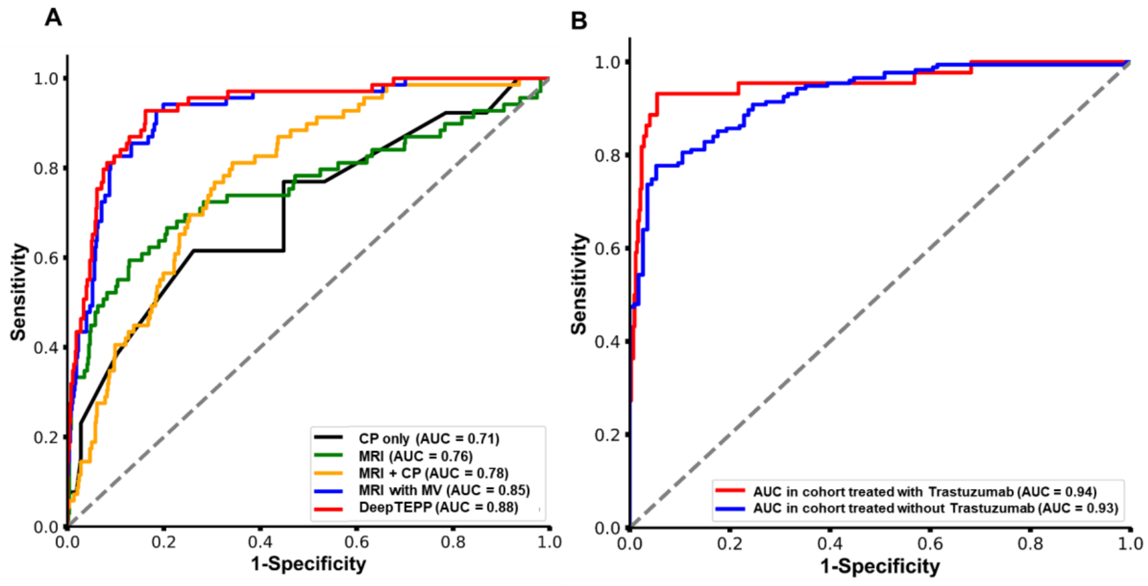

Figure S3. Predictive performance of DeepTEPP.

(A) Comparison of the predictive performance of different models including clinical-pathological features and/or MR imaging data augmentation in whole Cohort. (B) Predictive performance of DeepTEPP in population treated with (red) or without (blue) Trastuzumab.

Abbreviations: CP, clinical-pathological; AUC, area under curve; MR, magnetic resonance images; MV, multi-view augmentation.

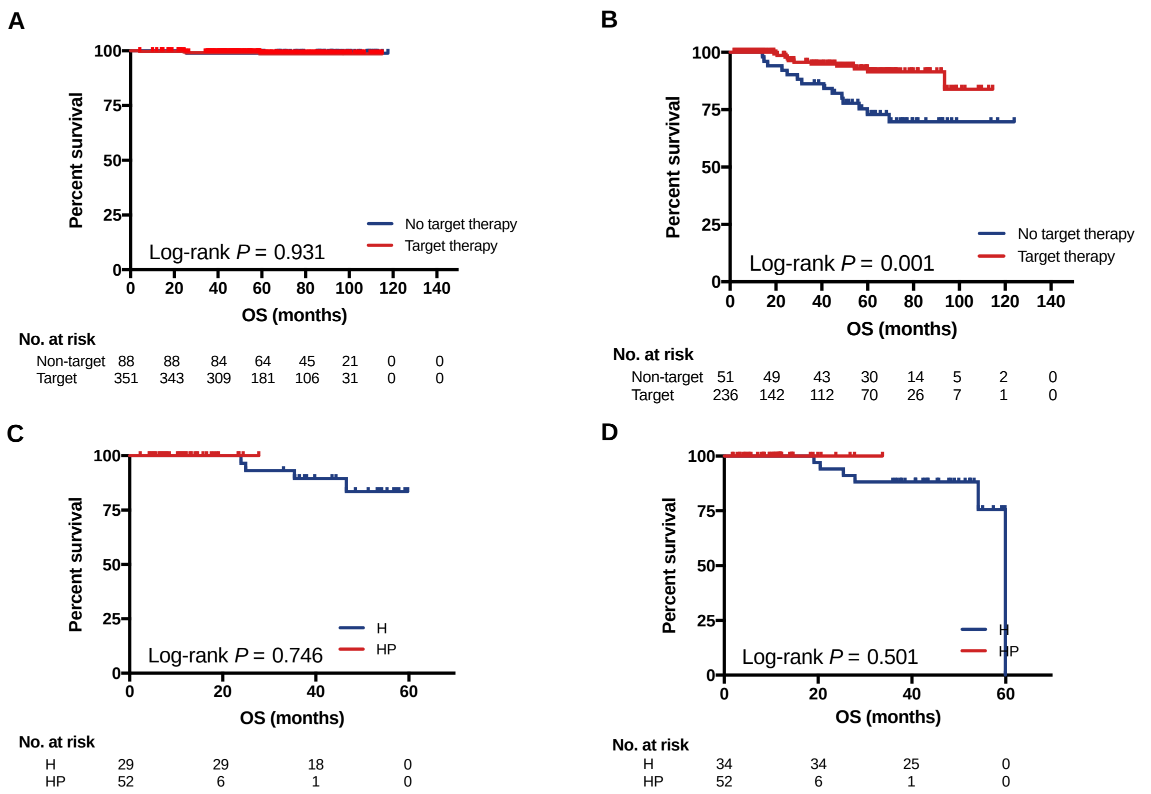

Figure S4. Overall survival by target therapy in different DeepTEPP risk groups.

(A) OS in DeepTEPP Low Cohort. (B) OS in high score patients. (C) OS in DeepTEPP Moderate patients. (D) OS in DeepTEPP High patients.

Abbreviations: OS, overall survival; H, Trastuzumab; P, Pertuzumab.

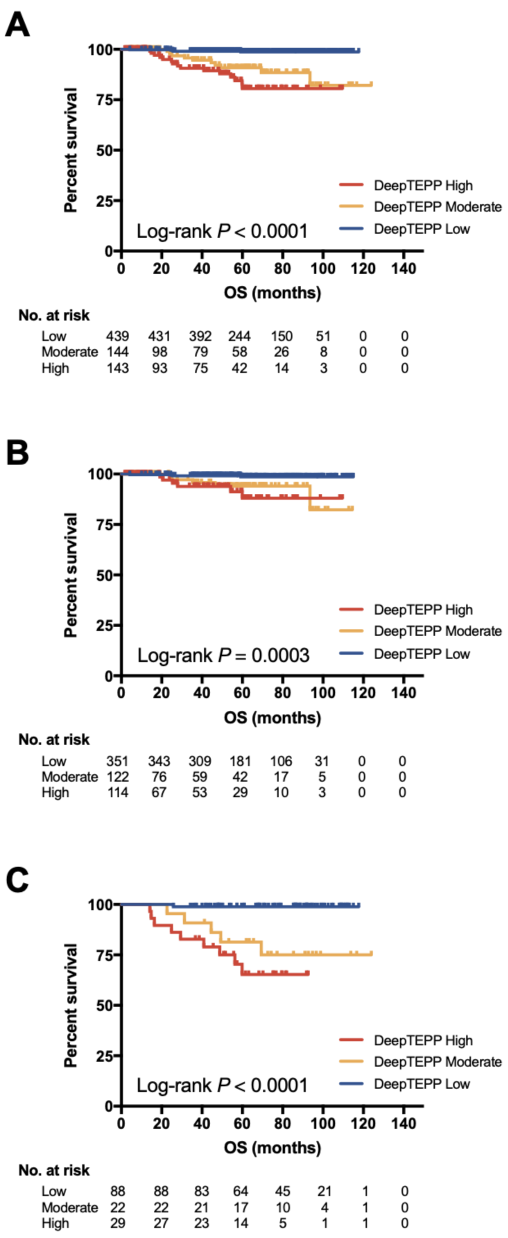

Figure S5. Prognostic value of DeepTEPP for overall survival. Overall survival according to DeepTEPP in the whole Cohort (A), in patients treated with anti-HER2 targeted therapy (B), and in those who didn't receive anti-HER2 treatment (C).

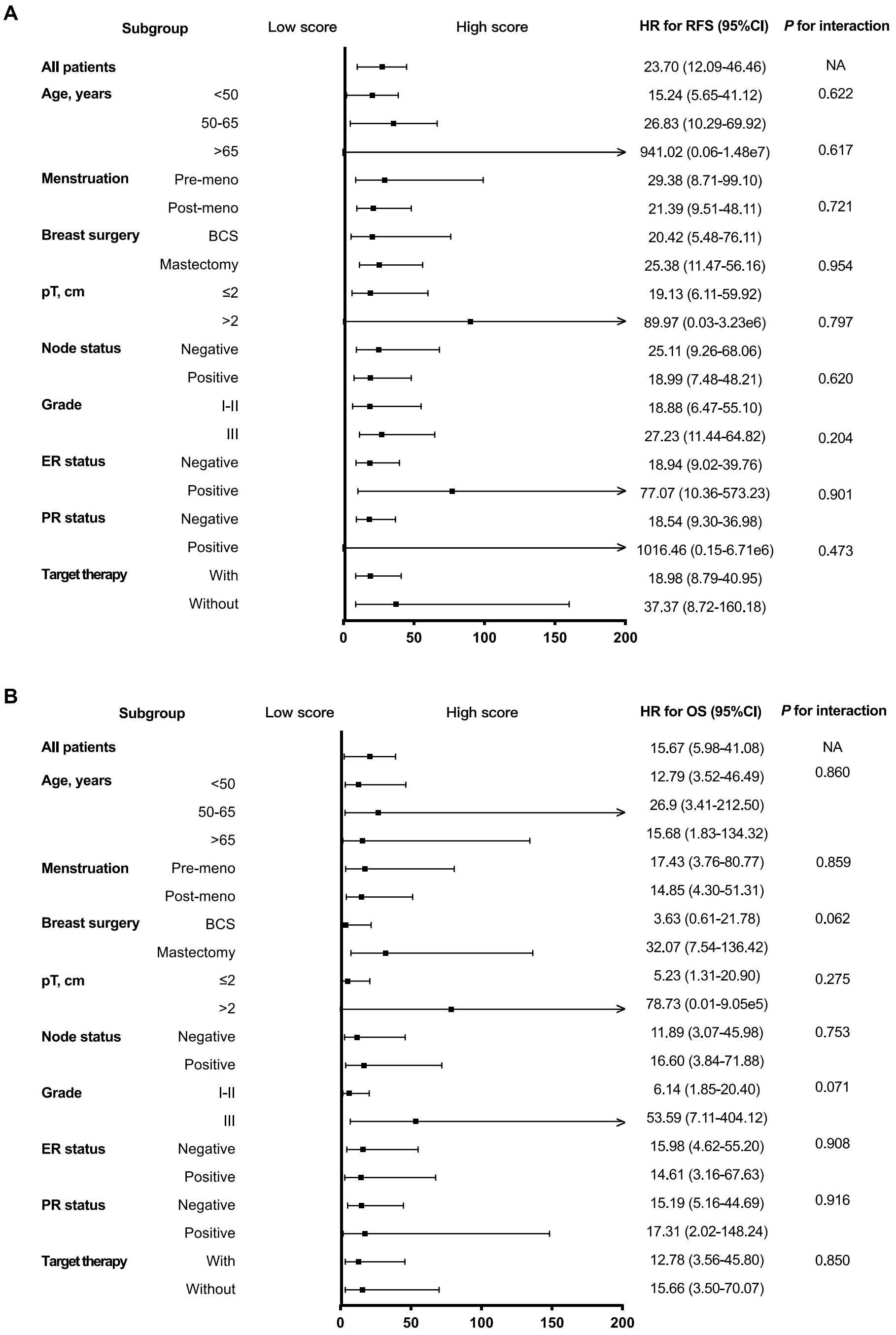

Figure S6. Subgroup analysis for the performance of DeepTEPP.

The prognostic performance of DeepTEPP on (A) RFS and (B) OS in different subgroups.

Abbreviations: HR, hazard ratio; RFS, recurrence-free survival; NA, not available; BCS, breast conserving surgery; ER, estrogen receptor; PR, progesterone receptor; OS, overall survival.

Table S1. Disease outcomes

Events	Total<br>N=726	No target therapy<br>N=139	H<br>N=468	H+P<br>N=119
**No event**	653 (89.94)	114 (82.01)	424 (90.60)	115 (96.64)
**Recurrence**	60 (8.26)	19 (13.67)	38 (8.12)	3 (2.52)
**Locoregional recurrence**	15 (2.07)	4 (2.88)	10 (2.14)	1 (0.84)
**Contralateral breast cancer**	13 (1.79)	6 (4.32)	7 (1.50)	0 (0.00)
**Distant metastasis**	32 (4.41)	9 (6.47)	21 (4.49)	2 (1.68)
**Breast cancer-specific death**	13 (1.79)	6 (4.32)	6 (1.28)	1 (0.84)

Table S2. Performance comparison of different networks

Networks	Dataset	AUC	ACC (%)	SPEC (%)	SENS (%)
**MM_ResNet (Ours)**	CV	0.76	89.79	96.65	36.23
	T	0.65	74.77	87.04	42.86
**VGG16**	CV	0.53	85.66	95.37	10.71
	T	0.51	66.52	92.04	7.47
**Desnet169**	CV	0.63	87.81	94.98	31.88
	T	0.62	85.82	93.87	28.86
**Shake**	CV	0.66	86.00	91.82	40.58
	T	0.61	88.77	96.90	26.19
**SE_ResNeXt**	CV	0.64	84.04	87.92	38.18
	T	0.58	78.71	87.55	32.54

Abbreviations: AUC, area under curve; ACC, accuracy; SPEC, specificity; SENS, sensitivity; CV, cross-validation; T, independent testing set.

Supplementary Table S3. Univariate survival analysis of clinical-pathological variables

Variables	P value	
	RFS	OS
**Age**	0.432	0.029
**Menstruation status**	0.428	0.976
**Breast surgery**	0.405	0.584
**Axillary surgery**	0.021	0.091
**Tumor size**	<0.001	0.003
**Node status**	<0.001	0.007
**Nuclear grade**	0.908	0.729
**ER status**	0.008	0.342
**PR status**	0.029	0.285
**Ki-67 (%)**	0.834	0.086
**Molecular subtype**	0.008	0.341

Abbreviations: RFS, recurrence-free survival; OS, overall survival; ER, estrogen receptor; PR, progesterone receptor.

Supplementary File S1

Method Details

1.1 Automatic tumor detection

Artificial breast tumor detection is often time-consuming and largely depends on the experience of experts. In this paper, a six-step automatic tumor detection algorithm was constructed (**Figure S1**). The automatic tumor segmentation was composed of a six-step algorithm, namely breast region cropping, binarization, erosion, dilation, central location selection, and screening of tumor location. First, images were cropped to reduce disturbances from non-breast tissues to the greatest extent. Then the image intensity was normalized from 0 to 255, and binarization was carried out adopting the threshold value of 130. After binarization, the noise was eliminated by erosion and dilation, where the elliptic erosion core was 3×3 and the elliptic dilation core was 8×3. After dilation, noise areas were eliminated by limiting the area to 100-1300. Since a small part of the heart was inevitably included in the ROI, which presented high signal as well, we proposed a new indicator named box density to distinguish tumors from other tissues. Box density is defined as follows: The sum of all the pixel values of the box divided by its area after the minimum bounding rectangle is reduced to half of the original in equal proportion, which is used to reflect the central brightness of the boxes. The mask with the highest box density is taken as the segmentation result. And then the minimum bounding rectangle of this mask was expanded by 1.2 times to retain the tumor edge information.

1.2 Multi-view data augmentation

In the training of MM_ResNet, recurrence status is used as the ground truth for assessing the risk level, and thus a risk score was obtained for each patient. However, the sample is highly unbalanced due to the fact that much fewer patients experienced recurrence (positive sample) than those without (negative sample), and the great imbalance with the ratio of 1:8 (positive: negative) can easily lead to the model focusing only on dominant group and not being adequately trained [1,2]. Therefore, we first solved this problem by implementing data augmentation, a commonly used medical image preprocessing methods [3,4]. In this paper we propose a new 3D data augmentation method, multi-view (MV) data augmentation, to solve this problem. Unlike conventional interpretation of MR images using only axial, coronal, and sagittal views, MV data augmentation samples the tumor ROI with different angles by simulating rotatable sampling planes, as shown in **Figure S2A**. For positive sample data, we set 8 times more sampling planes in 3D space than for negative sample data. After MV data augmentation, the positive and negative sample ratio changes from 1:8 to 1:1. For a given sampling plane, a scan parallel to the sampling plane is performed and the slices obtained from the scan are cyclically arranged into a matrix, i.e. a jigsaw, which is used as the network input.

1.3 Jigsaw shuffle strategy

Inspired by the Progressive Multi-Granulariy (PMG) network [5], the jigsaw inputs shown in **Figure S2A** are randomly shuffled by using a jigsaw puzzle generator, as shown in **Figure S2B**. The function of jigsaw puzzle generator is to obtain deformable convolution [6] without changing the convolution structure.

Deform convolution has more flexible receptive fields, which are used to capture global information, as shown in **Figure S2C**. The second row in the figure represents a point of the deep feature; the first row in the figure represents the receptive field of the point on the input image (blue solid arrow); the third row in the figure represents the receptive field mapped back to the scan sequence (black dotted arrow). It can be seen that the receptive field of all inputs in the first row is the same, but since the input is obtained after different shuffle filters, receptive field is also shuffled to extract more global features.

1.4 Network construction

We further introduce the network dubbed MM_ResNet, a multi-scale and multi-stage improved ResNet. As shown in **Figure S2D**, after obtaining four kinds of jigsaws, pretrained ResNet50 [7] is used as the backbone. The shallow layers of ResNet such as Conv1, Conv2, and Conv3 are used to extract low-level semantic features such as texture, grayscale, and edges of lesions. The deeper layers such as Conv4, Conv5 are used to extract deep semantic features that are ultimately beneficial for lesion classification. For the original jigsaw sequence, the features of multiple scales are fused to form more abundant features.

In the specific training process, we adopt progressive training strategy from the first stage to the fourth stage in turn [8]. Due to the limited receptive field and expression ability in the early stage, the network will be forced to first mine the information from low-level features (such as tumor texture, tumor edge, etc.) which can distinguish the status of recurrence. Compared with training the whole network directly, this strategy enables the network to learn gradually from low-order features to high-order features, instead of an upfront learning of all features. In final, at each stage we adopt cross entropy (CE) between ground truth label and prediction probability distribution for loss computation. After all four stages were trained, we optimized the sum of four losses so as to supervise the entire network.

1.5 Clinical-pathological information fusion

The fusion of image features and CP information can provide more valuable and optional features for the classifier and improve the prediction performance [9]. We include pathological tumor stage, lymph node status, estrogen receptor status, progesterone receptor status, and molecular subtype as CP features to improve MM_ResNet. The CP parameters are added by increasing the number of neurons and feeding CP directly into the penultimate layer of the fully connected layer of MM_ResNet [10]. We refer to MM_ResNet, which incorporates clinical information, as DeepTEPP.

1.6 Unsupervised K-Means clustering in the non-low risk group

After DeepTEPP is fully trained, each individual from the three Cohorts receives an output of risk score, and is then divided into low-risk group or non-low risk group ac-cording to the risk score cutoff with the highest Youden Index. And we analyze the extent to which patients in the low-risk group and non-low risk group benefited from anti-HER2 treatment. Because the non-low risk group is more required to decide on the regimen of targeted therapy, the DeepTEPP features of the patients in the non-low risk group were extracted and further clustered by using an unsupervised K-Means method. We cluster the network features incorporating CP information by K-means, which is expected to further differentiate patients into two

groups with different recurrence risks, i.e. moderate- and high-risk groups. The benefit of single and dual-targeted therapy was then further analyzed for both moderate- and high-risk groups.

Specifically, 1) The fusion feature before classifier 4 in **Figure S2** is used as the input of clustering; 2) Initialize the number of clustering categories as 2; 3) Always choose k-cluster centers at random; 4) Determine cluster mean or center; 5) Compute the difference between pixels and centers of each cluster; 6) If the distance to the center is near then transfer to that cluster; 7) Move to next cluster otherwise; 8) Re-evaluate the center; 9) Repeat the cycle before the core comes in. Parameter Settings are as follows: the K-Means algorithm will be run with different centroid seeds is 10, the maximum number of iterations of the K-Means algorithm for a single run is 3000, and relative tolerance with regards to inertia to declare convergence is 0.0001.

1.7 Implement details

Data Split Details: We divided Cohort 1 and Cohort 2 into five subsets sorted by patient names. In each iteration, we used a different subset as the validation set, and the remaining four subsets were used as the training set. We recorded the data paths, labels, patient names, and clinical indicators for Cohort 1 and Cohort 2 in a text file. The cross-validation set was then created using the KZDataset function in the train.py script. The decision to apply data augmentation was based on the resulting training set after the split.

Training Procedure: During each round of cross-validation, the network was trained for a fixed number of iterations (300) to ensure convergence and fit the data adequately. After training, five models were obtained, and each model predicted the corresponding test set samples from the held-out folds. The final predictions for each patient in Cohort 1 and Cohort 2 were obtained by combining the results from these five models. For independent testing on Cohort 3, we utilized the network that achieved the highest performance in the cross-validation phase.

Reason for training on Cohort 1+2 and testing on Cohort 3: In this paper, it is a common practice to train the model on a combined dataset consisting of Cohort 1, Cohort 2, and Cohort 3, and then evaluate its performance on a separate portion of the data. However, due to the availability of Pertuzumab for clinical use only starting from 2019, the follow-up time for Cohort 3 is significantly shorter compared to Cohorts 1 and 2, which may introduce inaccuracies in the ground truth labels. Including Cohort 3 in the training phase could potentially bias the model's optimization in the wrong direction. Therefore, Cohort 3 was not used for training purposes but served as an independent testing set to predict the scores.

References

1. Chiu TW, Tsai YL, Su SF. Automatic detect lung node with deep learning in segmentation and imbalance data labeling. Sci Rep-Uk 2021;11(1) doi ARTN 1117410.1038/s41598-021-90599-4.

2. Lin TY, Goyal P, Girshick R, He KM, Dollar P. Focal Loss for Dense Object Detection. Ieee I Conf Comp Vis 2017:2999-3007 doi 10.1109/Iccv.2017.324.

3. Long EP, Lin HT, Liu ZZ, et al. An artificial intelligence platform for the multihospital collaborative management of congenital cataracts. Nat Biomed Eng 2017;1(2) doi ARTN 002410.1038/s41551-016-0024.

4. Chen RJ, Lu MY, Chen TY, Williamson DFK, Mahmood F. Synthetic data in machine learning for medicine and healthcare. Nat Biomed Eng 2021;5(6):493-7 doi 10.1038/s41551-021-00751-8.

5. Du R, Chang D, Bhunia A, et al. Fine-Grained Visual Classification via Progressive Multi-granularity Training of Jigsaw Patches. 2020. p. 153-68.

6. Dai J, Qi H, Xiong Y, et al. Deformable Convolutional Networks. arXiv e-prints 2017:arXiv:1703.06211.

7. He K, Zhang X, Ren S, Sun J. Deep Residual Learning for Image Recognition. 2016 27-30 June 2016. p 770-8.

8. Du R, Chang D, Bhunia A, Xie J, Ma Z, Song Y-Z, et al. Fine-Grained Visual Classification via Progressive Multi-granularity Training of Jigsaw Patches. 2020. p. 153-68.

9. Duanmu H, Huang PB, Brahmavar S, et al. Prediction of Pathological Complete Response to Neoadjuvant Chemotherapy in Breast Cancer Using Deep Learning with Integrative Imaging, Molecular and Demographic Data. Medical Image Computing and Computer Assisted Intervention – MICCAI 2020; 2020; Cham. Springer International Publishing. p 242-52. (Medical Image Computing and Computer Assisted Intervention – MICCAI 2020).

10. Zheng X, Yao Z, Huang Y, et al. Deep learning radiomics can predict axillary lymph node status in early-stage breast cancer. Nat Commun 2020;11(1):1236 doi 10.1038/s41467-020-15027-z.
